# Non-Sustained Ventricular Tachycardia Episodes Predict Future Hospitalization in ICD Recipients with Heart Failure

**DOI:** 10.5935/abc.20170141

**Published:** 2017-10

**Authors:** Fatih Mehmet Uçar, Mustafa Adem Yilmaztepe, Gökay Taylan, Meryem Aktoz

**Affiliations:** Trakya University Hospital - Department of Cardiology, Turkey

**Keywords:** Heart Failure, Tachycardia, Ventricular, Defibrillators, Implantable, Hospitalization

## Abstract

**Background:**

Implantable cardioverter-defibrillator (ICD) therapy is well known to reduce mortality in selected patients with heart failure (HF).

**Objective:**

To investigate whether monitored episodes of non-sustained ventricular tachycardia (NSVT) might predict future HF hospitalizations in ICD recipients with HF.

**Methods:**

We examined 104 ICD recipients (mean age: 60 ± 10.1 years, 80.8 % male) with HF who were referred to our outpatient clinic for device follow-up. After device interrogation, patients were divided into NSVT positive and negative groups. The primary endpoint was the rate of hospitalization within the next 6 months after initial ICD evaluation.

**Results:**

Device evaluation demonstrated at least one episode of monitored NSVT in 50 out of 104 patients. As expected, no device therapy (shock or anti-tachycardia) was needed for such episodes. At 6 months, 24 patients were hospitalized due to acute decompensated HF. Hospitalization rate was significantly lower in the NSVT negative as compared with positive groups (38% versus 62%; adjusted hazard ratio [HR] 0.166 ; 95% CI 0.056 to 0.492; p = 0.01).

**Conclusions:**

Monitored NSVT bouts in ICD recordings may serve as a predictor of future HF hospitalizations in ICD recipients with HF suggesting optimization of therapeutic modalities in these patients along with a close supervision in the clinical setting.

## Introduction

Implantable cardioverter defibrillator (ICD) therapy has been regarded as the mainstay of sudden cardiac death (SCD) prevention among patients with HF, and significantly reduces overall mortality in these patients.^1,2^ In clinical practice, diminution of rehospitalizations in a given patient with HF serves as a predictor of favorable outcome, and may potentially mirror the optimality of therapeutic strategy as well. In line with this notion, ICD therapy was also suggested to be associated with lower HF readmission rates.^3^

Non-sustained ventricular tachycardia (NSVT) has been one of the most common challenges in clinical cardiology. It is generally defined as 3 or more consecutive beats arising below the atrioventricular node with a rate >120 beats/min and lasting less than 30 s.^4,5^ The ICD has treatment as well as monitorization options for NSVT. NSVT is associated with an increased risk for sustained tachyarrhythmia^5^ and is also a risk factor for SCD in patients with left ventricular dysfunction and hypertrophic cardiomyopathy.^6-8^ In other terms, NSVT is a common finding in Holter monitoring of patients with HF and is associated with a poor outcome.^9^ The present study aims to investigate the potential impact of NSVT episodes on the incidence of future HF hospitalizations among ICD recipients with HF.

## Methods

### Study Population and enrolment

**Figure 1:**
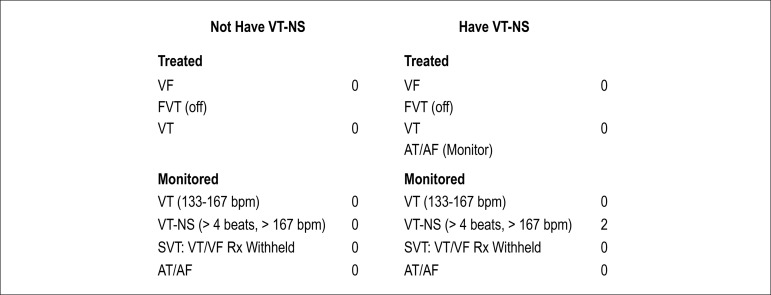
NSVT positive and negative definition of ICD record.

This observational prospective study was performed between November 2015 and May 2016 at Cardiology Clinic of the Trakya University Hospital, in Edirne, Turkey. ICD records contain data between previous index evaluation and the current day. Previous ICD follow-up of study patients were done 6 months before the study beginning day. NSVT was defined in monitored zone of ICD as 4 or more consecutive beats arising below the atrioventricular node with a rate > 167 beats/min and shorter than 16 beats (Figure 1). Patients who had NSVT episodes were defined as group-I and who had not any arrhythmia episodes were defined as group-II.

The patients with decompensated heart failure at the time of enrollment, atrial fibrillation-flutter, primary valvular pathology, advanced chronic obstructive pulmonary disease, recent infection, malignancy, blood dyscrasia, autoimmune or inflammatory disease, renal failure and hepatic failure were excluded from the study. Additionally, to discriminate ventricular arrhythmias and supraventricular arrhythmias more accurately, only patients with dual chamber ICD were selected, and patients receiving any ICD therapy (Shock or ATP) or monitored VT (133-167 bpm) were excluded from the study.

Information including age, gender, diabetes mellitus, hypertension and hyperlipidemia was gathered. The definition of HT was a systolic blood pressure (BP) value of ≥ 140 mmHg and/or a diastolic BP value of ≥ 90 mmHg at least on > 2 BP measurements or being on an antihypertensive therapy.^10^ The definition of DM comprised a blood sugar value of ≥ 126 mg/dl (7.0 mmol/l) in the fasting state or being on an antidiabetic therapy^11^ whereas the status of hyperlipidemia was based on the presence of a blood cholesterol level of ≥ 200 mg/dl or a triglyceride level of ≥ 150 mg/dl in the fasting state. The study was approved by the local ethics committee, and was implemented in complete concordance with the Declaration of Helsinki on human research. All subjects gave written informed consent to participate. 

### Follow-up and data collection

Implantable cardiac defibrillator interrogation was performed in the beginning of the study. All ICD’s zones were as VT (167-200 bpm) with discriminators and VF (> 200 bpm). Standard VT was defined as sustained tachycardia with a cycle interval ranging 300 to 360 msec. VF was defined as when the cycle interval was shorter than 300 msec. NSVT was defined as a regular rhythm wide complex tachycardia lasting four or more beats, higher rate than 167 bpm and shorter than 16 beats. Two independent electrophysiologists blinded to study design performed ICD interrogations, reviewed, and classified the arrhythmia episodes. When no consensus was reached, a third physician was included, and the final judgment was based on the majority decision.

At enrolment, a detailed patient history and the medications were noted. Echocardiography was performed for the evaluation of left ventricular ejection fraction, and the device follow-up results were collected in ICD follow-up unit. Clinical follow-up visits were scheduled at monthly. At each follow-up visit, the same physician blinded to the cause for the patient's presentation evaluated signs and symptoms of HF deterioration by auscultation and examination for leg edema and jugular vein distension. 

A chest X-ray was performed to detect signs of pulmonary congestion and when cardiac decompensation suspected, patient was admitted to inpatient clinic. 

### Statistical analysis

Continuous variables were expressed as mean (standard deviation) if the distribution was normal and as median (interquartile range) if the distribution was abnormal. The normality of distribution for continuous variables was confirmed with the Kolmogorov-Smirnov test. Categorical variables were expressed as number and percentages. A χ^2^ test or Fisher’s exact test was performed to compare the categorical variables. Non-paired student’s t-test or Mann- Whitney U test was used for continuous variables, as appropriate. Cox regression analysis was used to evaluate the relationship between variables and NSVT episodes. The results of the Cox analysis were presented as hazard ratios (HR) and 95% confidence intervals (CI). Receiver operating characteristic curve analysis was used to determine the optimum cutoff levels of the NSVT episodes to predict hospital admission. All statistical analyses were performed with SPSS software version 17.0 (SPSS Inc., Chicago, IL). A p value of 0.05 was considered statistically significant.

## Results

NSVT episodes were observed in 50 out of 104 patients (48 %) at the initial ICD evaluation. Study population were categorized into two subgroups if there were or not a NSVT episode (group I: 54 patients with NSVT and group II: 50 patients without NSVT). The baseline characteristics of the study population are shown in Table 1. Baseline characteristics were comparable between the two groups. The results of hematological and biochemical parameters are listed in Table 2. Laboratory parameters were also comparable between the groups.

**Table 1:** Baseline demographic and clinical features in ICD patients with and without NSVT

	Group I NSVT (-) (n = 54)	Group II NSVT (+) (n = 50)	p
Male, n (%)	(42) (77.7)	(42) (84.0)	0.42
Age (years, mean ± SD)	60 ± 10.1	61 ± 10.1	0.72
Hypertension, n (%)	25 (46)	24 (48)	0.86
Diabetes, n (%)	15 (27)	12 (24)	0.66
**Device**			
CRT, n (%)	11 (20)	6 (12)	0.24
ICD, n (%)	43 (80)	44(78)	
Ischemic Etiology, n (%)	25 (46)	30 (60)	0.16
Secondary Prevention, n (%)	21 (38)	17 (34)	0.60
Ejection Fraction (%)	28 ± 5.1	28 ± 5.7	0.98
Angiotensin-converting enzyme inhibitors, n (%)	42 (77)	40 (80)	0.78
Spironalactone, n (%)	29 (53)	34 (68)	0.13
Digoxin, n (%)	11 (20)	13 (26)	0.50
Diuretics, n (%)	30 (55)	35 (70)	0.13
Beta-blocker, n (%)	47 (87)	46 (92)	0.24
Statin, n (%)	27 (50)	28 (56)	0.56
Amiodarone, n (%)	7 (12)	2 (4)	0.10
Ivabradine, n (%)	8 (14)	8 (16)	0.86

NSVT: nonsustained ventricular tachycardia; ICD: implantable cardioverter-defibrillator; CRT: cardiac resynchronization therapy; SD: standart deviation.

**Tabela 2:** Comparison of biochemical and hematological characteristics and hospitalization in ICD patients with and without NSVT

	Group I NSVT (-) (n = 54)	Group II NSVT (+) (n = 50)	p
Glucose, mg/dL	124 ± 70.1	114 ± 40.1	0.40
Cratinine, mg/dL	1.01 ± 0.34	0.9 ± 0.24	0.63
Sodium, mg/dL	135 ± 17.3	137 ± 3.9	0.52
Potassium, mg/dL	4.5 ± 0.53	4.5 ± 0.57	0.98
Low-density lipoprotein, mg/dL	107 ± 39.9	106 ± 36.1	0.97
High-density lipoprotein, mg/dL	40 ± 12.4	38 ± 12.8	0.57
Asparate transaminase, mg/dL	28 (14-113)	26 (8-65)	0.53
Alanine transaminase, mg/dL	25 (5-115)	25 (3-71)	0.95
Hemoglobin, g/dL	12.9 ± 1.72	13 ± 2.04	0.82
Platelet, x 10^3^/L	244 ± 90.6	235 ± 63.6	0.54
White blood cell, x 10.9 /µl	8.1 ± 2.32	8,9 ± 3.02	0.14
TSH, mU/L	2.1 ± 1.75	2.2 ± 2.85	0.80
Free T3,ng/dL	2.5 ± 0.75	2.7 ± 0.81	0.31
Free T4,ng/dL	1.1 ± 0.32	1.1 ± 0.25	0.67
Hospitalization, n (%)	5 (9)	19 (38)	0.001

NSVT: nonsustained ventricular tachycardia; ICD: implantable cardioverter-defibrillator; HET: thyroid stimulant hormone.

**Figure 2:**
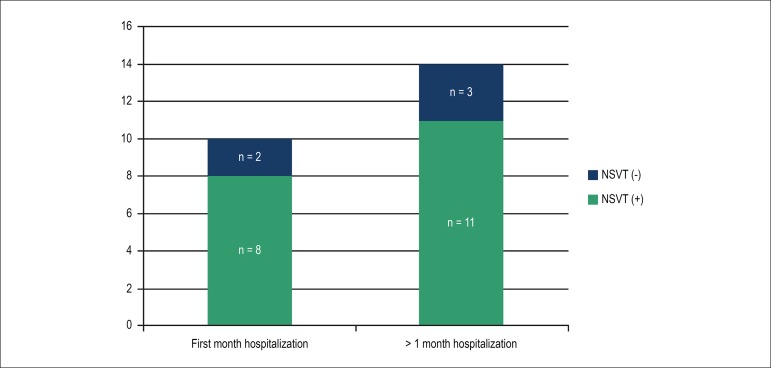
Time to hospital admission of study patients due to decompensation

**Figure 3:**
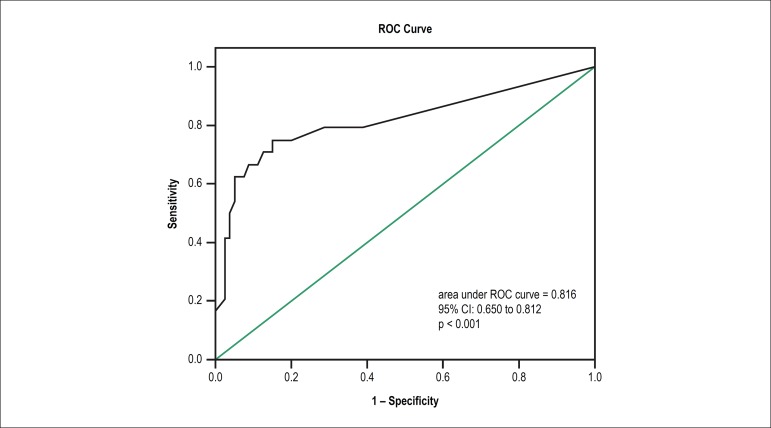
ROC curve analysis between hospitalization and non sustained ventricular tachycardia episodes

At 6 months following the initial ICD interrogation, 24 patients were eventually hospitalized due to HF decompensation. Hospitalization was significantly lower in the NSVT negative versus positive groups (38% versus 62%; adjusted hazard ratio [HR] 0.166 ; 95% CI 0.056 to 0.492; p = 0.01) (Table 2). Patients were rehospitalized due to HF more frequently within the first month as compared with the following months. Totally, 10 out of 24 hospitalized patients were admitted within the first month. Moreover, 8 out of these 10 were in group II (Figure 2). Analysis of receiver operating characteristic (ROC) for NSVT episodes (area under curve 0.816, 95% CI 0.650 to 0.812, p < 0.001) demonstrated that a total NSVT number of ≥ 19 had a strong discriminatory power to predict future HF hospitalization (Sensitivity 67%, Specificity 88%) (Figure 3).

## Discussion

The present study clearly demonstrates that monitored NSVT episodes in the initial ICD recordings appear to be associated with HF decompensation and re-hospitalization during the 6 months after the index evaluation with a predominantly higher rate of admissions within the first as compared with the following months.

Previous studies suggested NSVT as an important prognostic determinant for arrhythmic events.^12,13^ NSVT and frequent ventricular premature beats were previously shown to have a significant association with a higher arrhythmia risk in patients with dilated cardiomyopathy^14^ More importantly, NSVT is strongly associated with an increased SCD risk in the setting of hypertrophic cardiomyopathy.^8,15^ Even though the potential association of NSVT with further malignant arrhythmic events has been clarified to some extent, relationship between heart failure decompensation and NSVT is yet to be thoroughly elucidated.

Ventricular arrhythmias are frequently encountered in patients with HF^9^ with an overall incidence of NSVT ranging between % 30 and %80.^16,17^ NSVT is also common in ambulatory ECG recordings of HF patients and is associated with poor outcome.^9^ NSVT was suggested as an independent predictor of total mortality in patients with HF.^16^ Moreover, NSVT was found to be predictive for ICD-derived arrhythmias in patients with ischemic or nonischemic cardiomyopathy.^18^

Exact mechanisms linking NSVT to adverse outcomes remain unclear. One such mechanism for this association may be ascribed to sympathetic hyperactivation: During a NSVT episode, the blood pressure may fall drastically eliciting a subsequent sympathetic burst, which, in turn, might disturb cardiac structure and performance in the long term as a result of repetitive arrhythmic episodes ultimately leading to a state of progressive heart failure and cardiac decompensation.^19^

Secondly, increased sympathetic activity is a predictor of malign arrhythmias^20^ and a trigger of adverse myocardial remodeling. Accordingly, NSVT might be considered as a consequence of progressive myocardial failure associated with enhanced sympathetic activation or other triggers. In other words, an existing primary condition or abnormality manifesting as a progressive myocardial failure may ultimately predispose to malignant arrhythmias including NSVT. For exemple, electrical storm is an ominous finding in ICD recipients and is associated with worsening HF leading to an increased risk for sudden and non-sudden cardiac mortality.^21,22^

In the present study, we found a significant relationship between monitored NSVT episodes and hospitalization rates at 6 months. Our study has important clinical implications; Pacemakers are successful rhythm detection devices and ICD follow-up serves as an easy way to detect a long-term rhythm record of patients. NSVT detection in ICD recordings of patients with HF may be an important tool for the prediction of decompensated heart failure development in the near future. Rates of HF rehospitalization may be substantially diminished through close monitoring and optimization of medical therapy in these patients.

There are some limitations of the present study. This was a single-centre study, and included limited number of patients. Because of the sample size and inadequate power, it seems quite possible that some associations might have gone undetected. Moreover, the potential impact of other arrhythmias including PVCs was not taken into account. Further prospective studies are needed to substantiate the prognostic role of NSVT episodes in the prediction of future heart failure decompensation.

## Conclusion

Non-sustained ventricular tachycardia episodes may predict future heart failure decompensation in ICD recipients with HF. Detection of NSVT episodes in ICD recordings may entail optimization of medical therapy as well as close supervision of these patients in an effort to preclude future HF admissions.

## References

[1] Hua W, Niu H, Fan X, Ding L, Xu YZ, Wang J, ICD Study Group (2012). Preventive effectiveness of implantable cardioverter
defibrillator in reducing sudden cardiac death in the Chinese population: a
multicenter trial of ICD therapy versus non-ICD therapy. J Cardiovasc Electrophysiol.

[2] Moss AJ, Hall WJ, Cannom DS, Daubert JP, Higgins SL, Klein H (1996). Improved survival with an implanted defibrillator in patients
with coronary disease at high risk for ventricular arrhythmia. Multicenter
automatic defibrillator implantation trial investigators. N Eng J Med.

[3] Khazanie P, Hellkamp AS, Fonarow GC, Bhatt DL, Masoudi FA, Anstrom KJ (2015). Association between comorbidities and outcomes in heart failure
patients with and without an implantable cardioverter-defibrillator for
primary prevention. J Am Heart Assoc.

[4] Buxton AE, Duc J, Berger EE, Torres V (2000). Nonsustained ventricular tachycardia. Cardiol Clin.

[5] Katritsis DG, Siontis GC, Camm AJ (2013). Prognostic significance of ambulatory ecg monitoring for
ventricular arrhythmias. Prog Cardiovasc Dis.

[6] de Sousa MR, Morillo CA, Rabelo FT, Nogueira Filho AM, Ribeiro AL (2008). Non-sustained ventricular tachycardia as a predictor of sudden
cardiac death in patients with left ventricular dysfunction: a
meta-analysis. Eur J Heart Fail.

[7] Maron BJ, Savage DD, Wolfson JK, Epstein SE (1981). Prognostic significance of 24 hour ambulatory
electrocardiographic monitoring in patients with hypertrophic
cardiomyopathy: a prospective study. Am J Cardiol.

[8] Monserrat L, Elliott PM, Gimeno JR, Sharma S, Penas-Lado M, McKenna WJ (2003). Non-sustained ventricular tachycardia in hypertrophic
cardiomyopathy: an independent marker of sudden death risk in young
patients. J Am Coll Cardiol.

[9] Zipes DP, Camm AJ, Borggrefe M, Buxton AE, Chaitman B, Fromer M, American College of Cardiology, American Heart Association Task Force, European Society of Cardiology Committee for Practice
Guidelines, European Heart Rhythm Association, Heart Rhythm Society (2006). ACC/AHA/ESC 2006 Guidelines for Management of Patients With
Ventricular Arrhythmias and the Prevention of Sudden Cardiac Death:a report
of the American College of Cardiology/American Heart Association Task Force
and the European Society of Cardiology Committee for Practice Guidelines
(writing committee to develop Guidelines for Management of Patients With
Ventricular Arrhythmias and the Prevention of Sudden Cardiac Death):
developed in collaboration with the European Heart Rhythm Association and
the Heart Rhythm Society. Circulation.

[10] James PA, Oparil S, Carter BL, Cushman WC, Dennison-Himmelfarb C, Handler J (2014). 2014 evidence-based guideline for the management of high blood
pressure in adults: report from the panel members appointed to the Eighth
Joint National Committee (JNC 8). JAMA.

[11] American Diabetes Association (2010). Diagnosis and classification of diabetes mellitus. Diabetes Care.

[12] Multicenter Postinfarction Research Group (1983). Risk stratification and survival after myocardial
infarction. N Engl J Med.

[13] Mukharji J, Rude RE, Poole WK, Gustafson N, Thomas LJ Jr, Strauss HW (1984). Risk factors for sudden death after acute myocardial infarction:
Two-year follow-up. Am J Cardiol.

[14] Grimm W, Christ M, Bach J, Muller HH, Maisch B (2003). Noninvasive arrhythmia risk stratification in idiopathic dilated
cardiomyopathy: Results of the marburg cardiomyopathy study. Circulation.

[15] JR Gimeno, Tome-Esteban M, Lofiego C, Hurtado J, Pantazis A, Mist B (2009). Exercise-induced ventricular arrhythmias and risk of sudden
cardiac death in patients with hypertrophic cardiomyopathy. Eur Heart J.

[16] Doval HC, Nul DR, Grancelli HO, Varini SD, Soifer S, Corrado G, Gesica-GEMA investigators (1996). Nonsustained ventricular tachycardia in severe heart failure.
Independent marker of increased mortality due to sudden
death. Circulation.

[17] Singh SN, Fisher SG, Carson PE, Fletcher RD (1998). Prevalence and significance of nonsustained ventricular
tachycardia in patients with premature ventricular contractions and heart
failure treated with vasodilator therapy. Department of veterans affairs chf
stat investigators. J Am Coll Cardiol.

[18] Verma A, Sarak B, Kaplan AJ, Oosthuizen R, Beardsall M, Wulffhart Z (2010). Predictors of appropriate implantable cardioverter defibrillator
(icd) therapy in primary prevention patients with ischemic and nonischemic
cardiomyopathy. Pacing Clin Electrophysiol.

[19] Triposkiadis F, Karayannis G, Giamouzis G, Skoularigis J, Louridas G, Butler J (2009). The sympathetic nervous system in heart failure physiology,
pathophysiology, and clinical implications. J Am Coll Cardiol.

[20] Smith ML, Joglar JA, Wasmund SL, Carlson MD, Welch PJ, Hamdan MH (1999). Reflex control of sympathetic activity during simulated
ventricular tachycardia in humans. Circulation.

[21] Gatzoulis KA, Andrikopoulos GK, Apostolopoulos T, Sotiropoulos E, Zervopoulos G, Antoniou J (2005). Electrical storm is an independent predictor of adverse long-term
outcome in the era of implantable defibrillator therapy. Europace.

[22] Izquierdo M, Ruiz-Granell R, Ferrero A, Martinez A, Sanchez-Gomez J, Bonanad C (2012). Ablation or conservative management of electrical storm due to
monomorphic ventricular tachycardia: Differences in outcome. Europace.

